# Evolution of a plant sex chromosome driven by expanding pericentromeric recombination suppression

**DOI:** 10.1038/s41598-024-51153-0

**Published:** 2024-01-16

**Authors:** Dmitry A. Filatov

**Affiliations:** Department of Biology, University of Oxford, Oxford, OX1 3RB UK

**Keywords:** Evolution, Evolutionary genetics, Molecular evolution, Genome evolution

## Abstract

Recombination suppression around sex-determining gene(s) is a key step in evolution of sex chromosomes, but it is not well understood how it evolves. Recently evolved sex-linked regions offer an opportunity to understand the mechanisms of recombination cessation. This paper analyses such a region on *Silene latifolia* (Caryophyllaceae) sex chromosomes, where recombination was suppressed in the last 120 thousand years ("stratum 3"). Locating the boundaries of the stratum 3 in *S. latifolia* genome sequence revealed that this region is far larger than assumed previously—it is about 14 Mb long and includes 202 annotated genes. A gradient of X:Y divergence detected in the stratum 3, with divergence increasing proximally, indicates gradual recombination cessation, possibly caused by expansion of pericentromeric recombination suppression (PRS) into the pseudoautosomal region. Expansion of PRS was also the likely cause for the formation of the older stratum 2 on *S. latifolia* sex chromosomes. The role of PRS in sex chromosome evolution has been underappreciated, but it may be a significant factor, especially in the species with large chromosomes where PRS is often extensive.

## Introduction

Sex chromosomes evolved independently and re-evolved repeatedly^1^ in many groups of animals and plants^2,3^. Typically, Y- and X-chromosomes (or W- and Z-chromosomes in female heterogamety) originate from a single pair of autosomes and initially have the same gene composition. They acquire sex-determining (SD) gene(s), stop recombining around the SD gene(s) in the heterogametic sex^4^ and the non-recombining region gradually degenerates^2^, making X- and Y-chromosomes very different from each other.

Sex chromosomes provide a good illustration of the power of recombination to shape the properties of the genome. Formation of a non-recombining sex-specific region is a key step in evolution of sex chromosomes as it launches a cascade of events leading to the typical properties of sex chromosomes—genetically degenerate non-recombining Y-chromosome (NRY) and gene rich X-chromosome. Recombination (or lack of it) is one of the most important factors affecting evolutionary change^5,6^. It ensures that evolutionary fates of different mutations in the genome are independent from each other, allowing natural selection to work more efficiently to eliminate deleterious and fix advantageous mutations. Suppression of recombination in a genomic region makes natural selection inefficient and leads to gradual loss of functional genes and accumulation of ‘junk DNA’ such as mobile elements^7–9^. Recombination between the sex chromosomes in the heterogametic sex may continue in the pseudoautosomal region (PAR), preventing genetic degeneration and divergence between the X- and Y-chromosomes in that region. Previous work in animals and plants shed light on many aspects of sex chromosome evolution^2,3, 10, 11^. However, the key question—how recombination suppression evolves, is not well understood^10,12, 13^.

Expansion of NRY over time, leading to inclusion of a larger proportion of the chromosome into the non-recombining region was reported in many organisms (e.g. Refs.^14–16^). These expansions leave a characteristic signature of ‘evolutionary strata’—lower divergence between the X- and Y-chromosomes in regions that stopped recombining more recently compared to older non-recombining regions^14^. Various mechanisms, such as inversions, pre-existing recombination suppression^17^, heterochiasmy (difference in recombination rate between sexes)^18^, accumulation of repetitive DNA and heterochromatinization^19^ could contribute to recombination suppression and NRY expansion. The evolutionary processes leading to NRY expansions are actively disputed^13,20–22^. Such processes include sexual antagonism^23–25^, meiotic drive^26^, neutral X:Y-divergence^21,27–29^, heterozygosity advantage^30–32^, pericentromeric recombination suppression^33^ and dosage compensation^22^. Sexually antagonistic (SA) genes are thought to play an important role in NRY expansion^23–25^. If a gene is advantageous to males and detrimental to females, it is beneficial to make it male-specific by linking it to the Y-chromosome, which leads to recombination suppression and NRY expansion^23^. However, relatively little experimental evidence in support of this hypothesis is available^34^.

**Figure 1:**
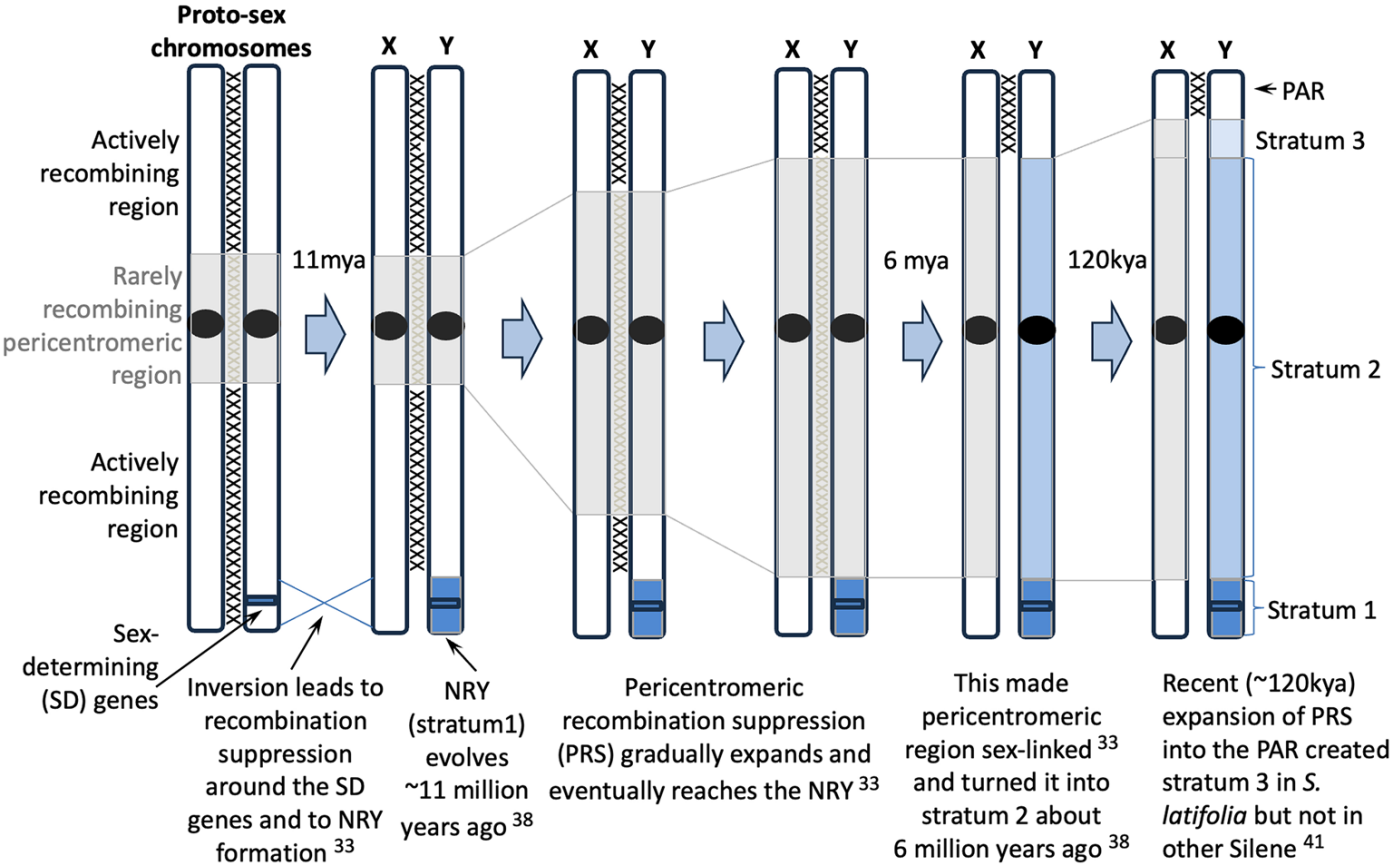
A possible scenario for evolution of recombination suppression on *S. latifolia* X and Y chromosomes. The XXX between the chromosomes show recombining regions, with grey XXX representing rarely recombining region. Blue shading shows male-specific non-recombining region on the Y chromosome, while grey shading shows the region with pericentromeric recombination suppression in both sexes.

*Silene latifolia* has been a classic model for studies of plan sex chromosomes (e.g.^35^) since 1923, when large cytologically distinguishable XX and XY chromosomes were reported in *S. latifolia* females and males, respectively^36^. Relatively recent origin of sex chromosomes in *S. latifolia* and its relatives offers an opportunity to study the mechanisms involved in evolution of recombination cessation between the X and Y chromosomes and the NRY expansion. Genus *Silene* includes over 700 species, most of which are non-dioecious and separate sexes and sex chromosomes are clearly derived traits^37^. Sex chromosomes in this lineage evolved only about 11 million years (MY) ago in the ancestor of *S. latifolia*^38^ from an ancestral autosome^39^, though translocations from other chromosomes to the sex chromosomes were also reported^40^. The NRY has formed and expanded in at least three steps, creating three evolutionary strata that are about 11, 6 and 0.12 MY old^16,33, 38, 41^. Based on the findings of the recent studies^33,41, 42^ I discuss the mechanisms of recombination cessation in the two older strata (Fig. 1) and then focus on the youngest stratum 3.

The *S. latifolia* female (XX) genome was recently sequenced, assembled to chromosome level and integrated with a genetic map^33^. This high-quality genome assembly revealed that the physical size of the stratum 1 is quite small (~ 15 Mb), indicating that recombination suppression on the nascent *S. latifolia* Y-chromosome has initially evolved in a relatively small region, similar to papaya^43^, persimmon^44^, kiwifruit^45^, *Asparagus*^46^ and *Ginkgo*^47^, though, in *S. latifolia* that region is actively recombining in females (green shading in Fig. 4 in reference^33^) unlike e.g. *Rumex*^17^. The stratum 1 includes sex determining gene SlCLV3 that acts as a gynoecium suppression factor (GSF) in males^48^ and it may have evolved recombination suppression to prevent recombination between GSF and the stamen promotion factor (SPF) that was located genetically on the Y chromosome^49^. Curiously, the order of genes in the stratum 1 is inverted in *S. latifolia* compared to a non-dioecious outgroup *Silene vulgaris*^33^, suggesting that the initial recombination suppression between the sex chromosomes in *S. latifolia* was caused by an inversion on the proto-X chromosome (Fig. 1). This finding goes against a widely held assumption in evolutionary genetic models that recombination suppression on the Y chromosome evolves due to inversions on the Y rather than the X^22,28, 50^. The X-linked inversions, remain to be taken into account in the evolutionary genetic models of NRY expansion.

NRY expansion ~ 6 million years (MY) ago^38^ created the stratum 2 that is physically very large (~ 330 Mb)^33^. Integration of genome sequence with female genetic map revealed extensive pericentromeric recombination suppression (PRS) in the stratum 2 of the X-chromosome in females, which could have contributed to NRY expansion^33^. An intriguing possibility is that stratum 2 evolved as a result of PRS expansion on the X chromosome^33^. According to this 'pericentromeric' model, stratum 2 evolved once the expanding pericentromeric region reached the stratum 1, which made the entire pericentromeric region sex-linked and turned previously pseudoautosomal pericentromeric region into the stratum 2 (Fig. 1). The expansion of the PRS on the X chromosome was revealed in comparisons of genetic maps of *S. latifolia* and *S. vulgaris*^33^. The physically massive pericentromeric region of the X chromosome is collapsed in *S. latifolia* male and female genetic maps due to lack of recombination in this region^42^, while in *S. vulgaris* map this region is well resolved and is actively recombining^33^, indicating PRS expansion on *S. latifolia* sex chromosomes. The role of PRS expansion in evolution of sex chromosomes (Fig. 1) has not been widely discussed in the literature and it deserves more attention of the research community.

The recent (< 0.12 MYA) evolution of the stratum 3 in *S. latifolia*^41^, represents the latest of the NRY expansions in *S. latifolia*. The previous analyses of this most recent NRY expansion were based on a few genes, with gene locations from a genetic map, as no chromosome-level genome assembly was available. In particular, six genes in the proximity of the PAR boundary, two of which were located in the stratum 3, were analysed by Campos et al.^51^, while Filatov^41^ analysed 16 genes from the stratum 3 and a number of genes in the adjacent regions. Here I take advantage of the recently published chromosome-level assembly of the *S. latifolia* genome^33^ to characterise it in the genome sequence and analyse the evolution of recombination suppression in this recently evolved stratum 3.

## Results

### Genomic positions of the PAR / stratum 3 / stratum 2 boundaries

The previous study^41^ analysed three regions in the proximity of the PAR boundary—the ‘left’ region located in the PAR, the ‘mid’ region (= stratum 3) sex-linked in *S. latifolia*, but not in other dioecious *Silene* and the ‘right’ region that is fully sex-linked in *S. latifolia* and its close relatives. For these regions 20, 16 and 21 genes were analysed, respectively^41^. Blast-searching the genes from the ‘left’, ‘mid’ and ‘right’ regions in the *S. latifolia* genome assembly^33^ identified their corresponding genomic locations and sizes. The pseudoautosomal genes from the ‘left’ region co-locate in the genomic region ~ 10 Mb long in the proximal part of the PAR. In total this 'left' genomic region contains 351 genes annotated in the genome assembly^33^. All the ‘mid’ genes are located in the genomic region adjacent to the PAR boundary. The 'mid' region is larger than assumed previously—it is over 5 Mb long and includes at least 125 genes. Unlike the ‘left’ and ‘mid’ genes that cluster together, the 21 previously analysed^41^ genes from the ‘right’ region are spread over 330 Mb of the stratum 2—the vast rarely recombining region on the X-chromosome that includes over 1000 other expressed genes. The order of genes assumed previously^41^ based on the genetic map^52^, is mostly consistent with the order in the genome for the ‘left’ and ‘mid’ regions, but not for the ‘right’ region that is poorly resolved in the genetic map due to lack of recombination in the central part of the X-chromosome in both sexes^33,42^. The availability of the genome sequence^33^ makes it possible to conduct more accurate analyses based on more genes with known locations in the regions adjacent to the PAR boundary.

To locate the PAR boundary in the genome sequence I followed the approaches described previously^41^, applying them to a larger set of genes annotated in the genomic sequence of the X-chromosome^33^. Segregation analysis in the *S. latifolia* genetic cross identified Y-linked SNPs in the gene Slati_XX0XG00010800 (gene ID 1080 in Table 1) and more proximally, in the genes with chromosomal positions > 24.843 Mb of the X-chromosome reference sequence. That segregation-based approach to identify Y-linked SNPs followed the methodology proposed previously^53^, except that the analysis here was done on individual progeny sequenced, while in the original study^53^ pools of male and female progeny were analysed. This approach was also used and accuracy of it confirmed in several other studies^41,52, 54, 55^. Segregation in a genetic cross cannot detect rare recombination events and the SNPs in the pseudoautosomal genes near the PAR boundary may appear Y-linked. Thus, the analysis in the genetic cross was complemented with the analysis of wild *S. latifolia* males and females to identify male-specific (= Y-linked) SNPs and locate the PAR boundary more precisely. Y-linked SNPs were detected in the gene Slati_XX0XG00011290 (gene ID 1129 in Table 1, located at position 27.082 Mb of the X-sequence reference) and the genes more proximally. Thus, the PAR in *S. latifolia* is ~ 27 Mb long, with about 1/3rd of it corresponding to the 'left' region analysed previously^41^.

**Table 1:** The location of boundaries between the PAR, stratum 3 and stratum 2.

Gene ID	Genome pos (kb)	Map pos (cM)		Stratum	Number of Y-SNPs			*K*_*s*_(XY)	Y-CDS length	
		Female	Male		Segr^a^	*S.lat*^b^	*S.dio*^c^		bp	%fullLen
1007	22,029	37.26	47.15	PAR						
1018	22,455	37.26	47.15	PAR						
1020	22,530	37.26	47.15	PAR						
1060	23,958	39.22	49.11	PAR						
1071	24,299	39.22	49.11	PAR						
1075	24,708	39.22	49.11	PAR						
1080	24,843	39.22	49.11	PAR	2			0.016	566	51
1098	25,582	39.22	49.11	PAR						
1103	25,942	39.22	49.11	PAR	1			0.01	411	54
1116	26,638	39.22	49.11	PAR	3			0.014	687	37
1125	26,872	39.22	49.11	PAR	1			0.012	393	65
PAR / stratum 3 border										
1129	27,082	39.22	49.11	Str3	2	1		0.027	459	93
1130	27,082			Str3	2	1		0.02	213	93
1133	27,326	39.22	49.11	Str3	7	4		0.048	1284	51
1134	27,333			Str3	2	2		0	162	58
1135	27,334	39.22	49.11	Str3	6	5		0.04	1086	92
1142	27,746	39.22	49.11	Str3	1			0	360	45
1143	27,774	39.22	49.11	Str3	11	5		0.016	1755	99
1144	27,819	39.22	49.11	Str3	11	5		0.032	1332	99
1146	27,853			Str3	5	2		0.037	861	75
1147	27,902			Str3	23	16		0.062	1272	98
1148	27,936	39.22	49.11	Str3	2	2		0.044	642	98
1151	27,956	39.22	49.11	Str3	4	3		0.025	1410	89
1153	28,108			Str3	10	4		0.056	815	98
1155	28,131			Str3	3	3		0.009	1044	69
1171	28,847			Str3	14	5		0.024	1383	67
1181	29,139			Str3	10	5		0.022	1620	74
1182	29,160			Str3	30	15		0.063	1929	99
1183	29,269	39.22	49.11	Str3	35	18		0.079	2709	95
1184	29,275	39.22	49.11	Str3	15	12		0.096	1662	97
1186	29,286	39.22	49.11	Str3	8	6		0.066	530	99
1188	29,293			Str3	8	3		0.067	501	99
1189	29,294			Str3	3	2		0.02	210	88
1190	29,342	39.22	49.11	Str3	6			0.074	489	84
1191	29,344	39.22	49.11	Str3	4	4		0.031	420	43
1192	29,403			Str3	2			0.031	192	72
1196	29,597			Str3	7	5		0.038	789	65
1200	29,769	39.22	49.11	Str3	7	7		0.037	993	98
1201	29,822	39.22	49.11	Str3	8	4		0.02	1323	64
1204	29,976	39.22	49.11	Str3	11	10		0.017	1350	95
1208	30,261	39.22	49.11	Str3	2	2		0.044	312	99
1210	30,273	39.22	49.11	Str3	7	5		0.059	1424	99
1215	30,457			Str3	9	7		0.052	1050	91
1222	30,591			Str3	4	2		0.11	659	93
1227	30,628			Str3	3	3		0.063	435	33
1228	30,654	39.22	49.11	Str3	22	11		0.049	1560	98
1241	31,320	39.22	49.11	Str3	21	21		0.051	1601	99
1242	31,349	39.22	49.11	Str3	10	5		0.086	1238	99
1243	31,352	39.22	49.11	Str3	5	1		0.03	714	91
1244	31,358			Str3	1			0.052	372	51
1245	31,381	39.22	49.11	Str3	2	1		0.05	474	34
1249	31,612			Str3	10	6		0.144	705	53
1258	31,855			Str3	7	5		0.073	588	90
1259	31,858			Str3	2	2		0.061	290	99
1266	32,920	39.22	49.11	Str3	1	1		0.074	195	54
1277	34,083	39.22	49.11	Str3	4	4		0.171	324	42
1285	34,674			Str3	5	4		0.054	635	87
Stratum 3 / stratum 2 border										
1331	41,574			Str2	32	32	19	0.044	2063	99
1335	41,897			Str2	13	13	5	0.026	980	92
1341	42,361		49.11	Str2	17	12	1	0.03	2238	84
1342	42,372	39.22	49.11	Str2	3	3		0.046	521	99
1352	43,093	39.22	49.11	Str2	2	1		0.088	929	81
1359	43,807	39.22	49.11	Str2	11	6	5	0.05	2390	99
1364	44,614	39.22	49.11	Str2	3	2	2	0.067	572	67

^a^Number of Y-SNPs identified with segregation analysis in *S. latifolia.*

^b^Number of Y-SNPs in *S. latifolia* identified in polymorphism analysis.

^c^Number of Y-SNPs in *S. dioica* identified in polymorphism analysis.

**Figure 2:**
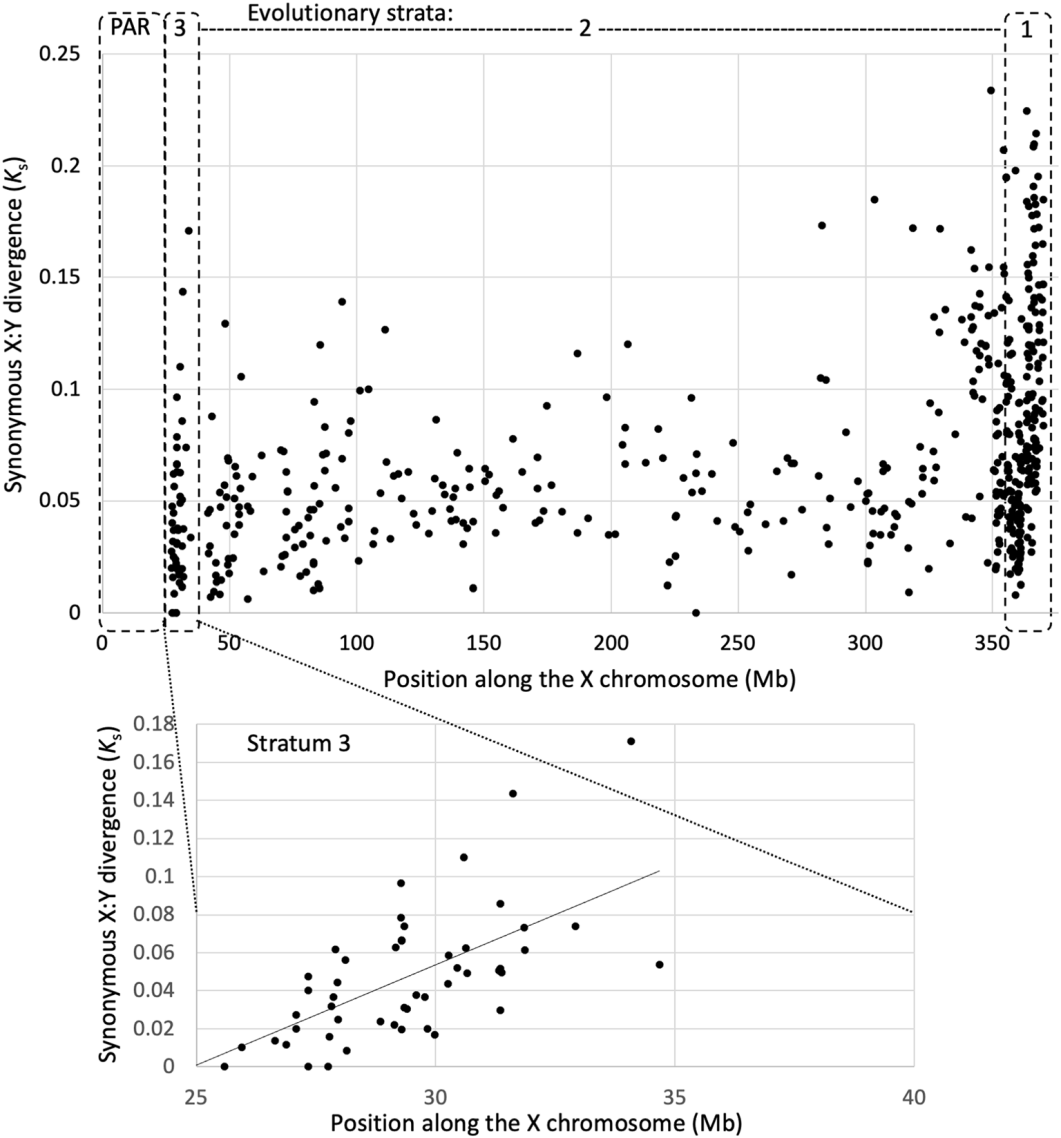
Synonymous sequence divergence between *S. latifolia* X- and Y-linked gametologs.

**Figure 3:**
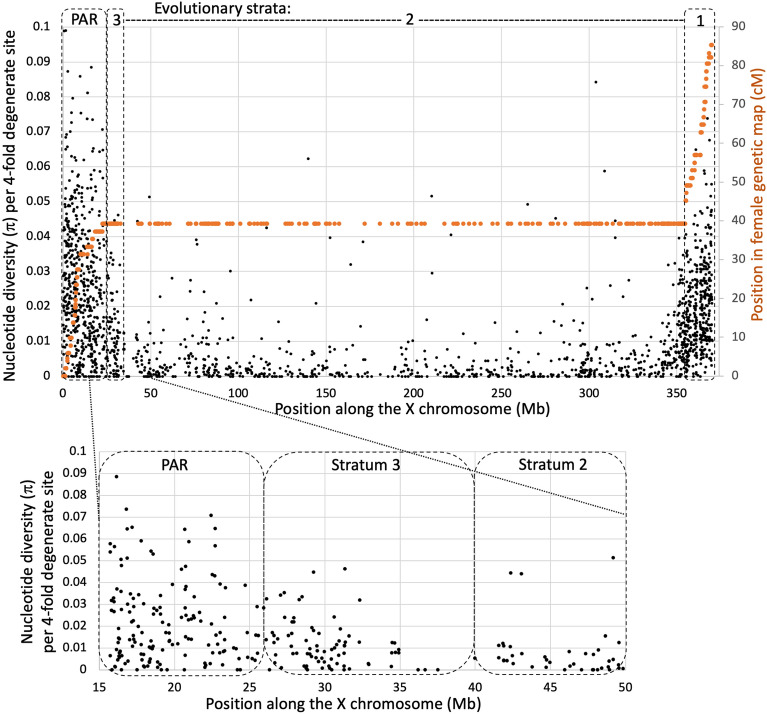
Synonymous genetic diversity on the X chromosome in *S. latifolia* females. Only fourfold degenerate codon positions were used in this analysis. Black points show per-nucleotide π in females, while the orange points show genetic position of markers in the female genetic map^42^.

In order to locate the boundary between the strata 2 and 3, the male-specific SNPs were analysed in *S. dioica—*a dioecious close relative of *S. latifolia* where the region corresponding to the stratum 3 is pseudoautosomal^41^. This revealed the presence of numerous Y-linked SNPs in *S. dioica* in the gene Slati_XX0XG00013310 (gene ID 1331 in Table 1) and more proximally, indicating sex-linkage of *S. dioica* genes proximally to the genomic position 41.5 Mb. Assuming the strata 2/3 boundary at position 41 Mb, stratum 3 in *S. latifolia* is 14 Mb long and contains 202 annotated genes. Thus, the stratum 3 is considerably bigger than ~ 1 Mb assumed previously^41^. The 41 genes annotated in the genome reference^33^ between 34.7 and 41.5 Mb are weakly expressed (FPKM < 1) and are uninformative in the transcriptome sequence-based analyses presented below. Thus, this region looks empty on Figs. 2, 3 and Table 1. It is not clear why this region is enriched for genes with low expression. Table 1 lists the stratum 3 genes where the Y-linked SNPs were found. In addition to that it lists a few adjacent PAR and stratum 2 genes to show the likely positions of the boundaries between the strata and the PAR.

### X:Y sequence divergence

The distribution of X:Y divergence in the stratum 3 and in the adjacent region could be informative about the mechanism of recombination cessation between the X and Y chromosomes in this region. As the sequence of the *S. latifolia* Y-chromosome is not yet publicly available, I reconstructed the sequences of Y-linked gametologs for the X-linked genes using the previously published approach^53^ based on separation of sequence reads containing Y-linked SNPs and their assembly into contigs (see Methods). This approach yielded partial sequences of coding regions for Y-linked gametologs of 540 genes annotated on the X chromosome. The average coding sequence (CDS) length of the assembled Y-linked sequences was 82.78% of the CDS length of the corresponding gametologs annotated on the X chromosome. In the stratum 3, reconstruction of the Y-linked sequence was possible only for 46 genes (Table 1). The average CDS completeness for the Y-linked stratum 3 genes was 80.6%. As expected, the X:Y synonymous divergence (Fig. 2) was the highest in the stratum 1 (mean *K*_s_ = 0.093 ± 0.0042 [standard error]), intermediate in the stratum 2 (mean *K*_s_ = 0.064 ± 0.0022) and the lowest in the stratum 3 (mean *K*_s_ = 0.043 ± 0.0051). It is interesting that the X:Y divergence near the PAR boundary is the lowest and increases proximally, forming a visible gradient (Fig. 2), which is suggestive of a gradual X:Y recombination cessation, as discussed below.

### Genetic diversity in the stratum 3 and the X chromosome

As the recombination suppression in the stratum 3 occurred very recently, this event may have left a trace in DNA sequence polymorphism. For example, if the factor causing X:Y recombination cessation is X-linked, such as an X-linked inversion, it must have led to a reduction in genetic diversity on the X chromosome in the stratum 3 due to a selective sweep caused by spread of the inversion across the *S. latifolia* population. On the other hand, a Y-linked inversion would not cause reduction in genetic diversity on the X and its effect on the Y would also be negligible because the Y has reduced diversity^56^. The average synonymous nucleotide diversity values in the PAR and strata 3, 2 and 1 are 0.026 ± 0.0008 (± standard error), 0.011 ± 0.0013, 0.007 ± 0.0004 and 0.022 ± 0.0008, respectively. No reduction of genetic diversity in detectable in the stratum 3 compared to the adjacent stratum 2 (Fig. 3), and no recent selective sweep is apparent in this region.

To place the stratum 3 genetic diversity in a wider context, the distribution of sequence polymorphism along the X chromosome is shown in Fig. 3. Genetic diversity for the sex-linked genes in males may be inflated by X:Y divergence for genes with X- and Y-linked gametologs, while genetic diversity in females reflects the actual polymorphism on the X-chromosome. The distribution of polymorphism along the X-chromosome in females is quite uneven, showing distinct peaks of genetic diversity in the PAR and stratum 1, while most genes in the strata 2 and 3 show limited level of polymorphism (Fig. 3). Rarely recombining regions, such as the stratum 2, are known to typically have reduced diversity compared to actively recombining parts of the genome^57^. This is thought to reflect stronger linked selection in rarely recombining regions where linkage disequilibrium is extensive and selection at one gene can affect genetic diversity in a large region around it^58^. Female recombination rate in the stratum 3 is also low, as indicated by lack of recombination in the genetic map in this region (Table 1). Consistent with this, linkage disequilibrium, measured with Z_nS_ statistic^59^ in *S. latifolia* wild females is high in both strata 2 and 3 (average Z_nS_ = 0.22 in both strata 2 and 3), while it is much lower (average Z_nS_ = 0.14) in the PAR genes in the 5 Mb adjacent to the PAR boundary. Lack of recombination in the strata 2 and 3 explains lower genetic diversity in these regions compared to actively recombining stratum 1 and the PAR.

## Discussion

This study reveals the genomic location of the recently evolved *S. latifolia* stratum 3, which enables the analyses of the processes driving the NRY expansion. The results presented above significantly extend the conclusions of the previous study^41^. The addition of many more genes to the analysis, as well as the information about the genomic locations of these genes, revealed that the expansion of the *S. latifolia* NRY was more extensive than assumed previously.

How did the recombination suppression in the stratum 3 evolve? While the exact mechanistic causes of the PAR boundary shift that created stratum 3 in *S. latifolia* are unclear, the distribution of X:Y divergence in the stratum 3 and in the adjacent region sheds some light on this process. A step-wise NRY expansion, e.g. caused by an inversion, would create a sharp boundary with a step increase in X:Y divergence between the new and the old strata. No such step increase in X:Y divergence is apparent at the border between the strata 2 and 3 in *S. latifolia* (Fig. 2). Furthermore, if the inversion that caused NRY expansion occurred on the X chromosome, this would move a block of the old stratum to the new PAR boundary, which should create a peak of higher X:Y divergence at the new PAR boundary (Fig. 1b in ref.^60^). However, an inversion on the Y-chromosome is not expected to create such a peak because the order of genes corresponds to that on the X chromosome in the female genome. The observed distribution of synonymous X:Y divergence does not reveal a detectable peak of X:Y divergence at the boundary between the stratum 3 and the PAR (Fig. 2). In fact, the X:Y divergence near the PAR boundary is the lowest and increases proximally, forming a visible gradient (Fig. 2), which is suggestive of a gradual X:Y recombination cessation, e.g. due to hindered chromosome pairing near the NRY^21^.

Multiple consecutive inversions on the Y across the PAR boundary represent a possible mechanism for the apparently gradual recombination suppression in the stratum 3. If each such inversion includes only one or few PAR genes, recombination suppression would expand into the PAR in small steps, leading to the gradient of X:Y divergence observed in the stratum 3. The spread and fixation of inversions extending NRY has been modelled^32,50, 61^, which revealed a bias towards preferential fixation of smaller inversions, expanding NRY in small steps that may look like a gradient, as on Fig. 2. However, it is not clear whether this process is fast enough for multiple inversions to gradually extend *S. latifolia* NRY in just 0.12MY. As this process is expected to scatter stratum 3 genes across the Y chromosome, co-location of stratum 3 genes to the same region of the Y chromosome and preservation of their order on the X and the Y would refute the involvement of multiple consecutive inversions across the PAR boundary in recombination suppression in stratum 3. The sequence of the *S. latifolia* Y chromosome, once publicly available, will allow one to test this hypothesis.

A gradual expansion of pericentromeric recombination suppression (PRS) into the PAR may be another mechanism for stratum 3 formation. Indeed, in the female genetic map^42^ the genetic markers in the stratum 3 have the same genetic position as the markers in the stratum 2 (all at position 39.22 cM, i.e. completely linked; Table 1), while the markers in the PAR with genomic positions < 23 Mb are recombining in both male and female genetic maps. This indicates that the current PAR boundary in *S. latifolia* approximately coincides with the boundary of the region where recombination is suppressed in both sexes. To test this, it would be interesting to analyse the *S. dioica* male and female recombination rates in this region. If the recent PAR boundary shift in *S. latifolia* was caused by PRS expansion, this region is expected to recombine more actively in *S. dioica* females compared to *S. latifolia* females. Unfortunately, no detailed enough genetic map is available for *S. dioica* to test this prediction. PRS expansion was hypothesized to be the cause of stratum 2 formation^33^ (Fig. 1). The absence of (or very rare) recombination in *S. latifolia* females at the strata 1/2 and stratum 3/PAR boundaries is the critical prediction of this model that can be tested with a high-resolution female *S. latifolia* map. The data in hand^33,41, 42^ indicate that this prediction is correct, but higher resolution maps from several independent genetic crosses would be needed to test this prediction of the PRS model more rigorously.

Expansion of PRS may be caused by various mechanisms, e.g. proliferation of transposable elements, or mechanistic constraints, such as postulated by the ‘telomere-initiation’ model^62,63^. Translocations to the ends of the chromosome may also play a role in PRS expansion—if chiasmata tend to form and recombination tends to occur close to the telomeres, then adding genetic material to the end of a chromosome should automatically shift previously telomeric region more proximally where recombination is less frequent. If PRS expansion is indeed involved in recombination suppression in both the strata 2 and 3, this mechanism may prove to play a major role in NRY expansion, at least in *S. latifolia*, and possibly more generally in plants where extensive recombination suppression at the central chromosome regions is common, especially in species with large chromosomes^62,64^.

## Methods

### Finding the locations of previously identified sex-linked genes in the genome

In order to find the genomic locations for previously identified genes near the PAR boundary the sequences of coding regions of these genes were blast-searched against the assembly of the *S. latifolia* female genome^33^. The fasta file with genome sequence was formatted into a blast database with formatdb program. CDS sequence for the genes of interest were blast-searched against that database using blastn program with options "-m 8 -e 0.000001". The resulting table listing blast hits was filtered to remove low identity (< 80%) and short (< 50b) hits.

### Transcriptome sequence data and SNP calling

The analyses in this study are based on transcriptome sequence data from 14 *S. latifolia* and 14 *S. dioica* wild individuals of both sexes as well as 55 males and females from a genetic cross (Table S1). The RNA for these RNA-seq datasets was extracted from actively growing shoots. An obvious limitation of this approach is that it limits the analysis to the genes expressed in these tissues. Transcriptome sequence data were mapped to coding sequences (CDS) from the *S. latifolia* female reference genome^33^. Read mapping was done with BWA mem 0.7.17^65^ with minimum seed length (-k) = 19, matching score (-A) = 1, mismatch penalty (-B) = 4, gap open penalty (-G) = 6 and gap extension penalty (-E) = 1. The mapped reads were passed through samblaster^67^ with parameters --excludeDups, --addMateTags, --maxSplitCount 2, --minNonOverlap 20 and then sorted and indexed with Samtools 1.7^66^. The total and aligned numbers of reads listed in Table S1 were obtained with commands samtools view -c bamFile.bam and samtools view -c -F 260 bamFile.bam, respectively. Then, SNP calling was done with samtools mpileup (options: -d 1000 -q 20 -Q 20) and bcftools 1.7^68^ call (options: -m -O v -g 8) and filter (options: -saFilter -g3 -G10 -e'%QUAL < 10 || (AC < 2 && %QUAL < 15) || FMT/DP < 5 || (GT = "0/1" && DP4[0]  + DP4[1]  < 2) ' -sHighDepth -e '%MAX(DP) > 2000). The resulting multisample VCF file was converted to fasta alignments using ProSeq software^69^ available from https://sourceforge.net/projects/proseq/.

### Polymorphism analyses

Fasta alignments from VCF files (as described above) were imported in ProSeq4^69^ and coding sequence information was assigned to each sequence alignment with "Entire seq is CDS" menu item in ProSeq4. The accuracy of CDS assignment was checked with the "Coding regions report" tool in ProSeq4. Any CDS mis-assignments create numerous premature stop codons and thus easily detectable in this analysis. The same program was used to analyse DNA sequence polymorphism in *S. latifolia* males and females. To focus the analysis on neutral sites, the alignments were filtered to leave only fourfold degenerate codon positions, using "Filter sites in all datasets" tool in ProSeq4. Sequence polymorphism at these sites was summarised with ‘Analyse DNA polymorphism’ tool in ProSeq4.

### Identification of Y-linked SNPs

Y-linked SNPs were used to (i) locate the boundaries between the PAR and the strata and (ii) to reconstruct the sequences of Y-linked gametologs for the X-linked genes annotated in the *S. latifolia* genome assembly. Y-linked SNPs were identified in two ways. Firstly, the segregation analysis in the *S. latifolia* genetic cross was used to identify the SNPs that are inherited from father to sons and never to daughters. In this analysis transcriptome sequence data from 21 male and 32 female progenies of the genetic cross (Table S1), as well as from their parents and grandparents were used. Secondly, the transcriptome sequence data from a sample of unrelated wild *S. latifolia* and *S. dioica* male and female individuals was used to identify male-specific SNPs. For each of the species I used sequences from seven males and seven females (Table S1). The transcriptome data were mapped to CDS of genes extracted from the female reference genome^33^ and SNPs called as describe above. Only the SNPs with quality > 500 were retained for analysis. The following awk script was used to select the SNPs that are homozygous for the reference allele in the mother and heterozygous in the father of the genetic cross: “awk ‘{if (($10 ~ “0/1”)&&($11 ~ “0/0”)) print}’ allSNPs.vcf > filteredSNPs.vcf”, where the fields “$10” and “$11” correspond to SNP calls in the father and the mother individuals, respectively. The resulting filtered VCF file was small enough to be handled in Microsoft Excel, where the filtering for male-specific SNPs was done for the genetic cross and wild *S. latifolia* and *S. dioica*. Filtering in the genetic cross data required SNPs to have missing data in less than 10 progeny, to be absent in females and present in at least 10 male progeny. In the wild *S. latifolia* and *S. dioica* individuals the putative male-specific SNPs were required to be present in at least six males and none of the females from the same species.

### Assembly of the Y-linked genes

Reconstruction of sequences for the Y-linked gametologs for the X-linked genes were conducted as previously^53^. This approach is based on separation of sequence reads containing Y-linked SNPs and their assembly into contigs. Y-linked SNPs were identified as described above. A VCF file with these Y-SNPs was used to filter SAM files to separate sequence reads containing these Y-SNPs. For this purpose, the BAM file with sequence reads was converted to SAM format with samtools view. The resulting SAM file and the VCF file with Y-SNPs were used as input for program filterSAMbyVCF (available from https://sourceforge.net/projects/filtersambyvcf/) to separate the Y-specific sequence reads. That software identifies and outputs the reads containing non-reference ('ALT' field in VCF file) allele for the SNPs in the VCF file used as input for filterSAMbyVCF program. The SAM files with these Y-reads were loaded into ProSeq4 software^69^ that was used to call consensus sequence for each Y-linked gene with Y-reads available. The Y-consensus along with the Y-reads for each gene were visually checked in ProSeq4. The same program was used to align and compare the resulting Y-consensus sequences with their X-linked gametologs. The X:Y alignments were analysed for completeness of Y-CDS compared to X-CDS for each gene with "Gaps and missing data report" in ProSeq4. As the entire length of each alignment corresponded to coding sequence (as annotated in the reference genome^33^), the coding sequence information was assigned to each alignment with "Entire seq is CDS" menu item in ProSeq4. After assigning coding sequence information, the presence of premature stop codons was checked invoking the "Coding regions report" menu item in ProSeq4. The X:Y alignments with assigned coding sequence were used to calculate pairwise synonymous and non-synonymous sequence divergence in ProSeq4.

### Supplementary Information

Supplementary Table S1.

## Data Availability

The data used in this paper (along with the accession numbers) are listed in Table S1.
