# Supplementary material for: Evolution of a plant sex chromosome driven by expanding pericentromeric recombination suppression
Source: Sci Rep. 2024 Jan 16;14:1373. doi: 10.1038/s41598-024-51153-0 (PMC10791620; doi:10.1038/s41598-024-51153-0)
Supplement: Supplementary file 1 — Supplementary Table S1. [file 41598_2024_51153_MOESM1_ESM.pdf]

Supplementary Information

Evolution of a plant sex chromosome
driven by expanding pericentromeric recombination suppression.

Dmitry A. Filatov
Department of Biology, University of Oxford, Oxford, OX1 3RB, United Kingdom

Table S1. Transcriptome sequence data used in the analyses.

Name	Sex	Origin	Accession number	Read length	Median quality	Reads all	Reads aligned	Ref.
S. latifolia								
fSa985	f	Austria	SRX1100232	125	37	30,497,923	24,496,518	1
fSa1179	f	England, UK	SRR18750215	100	38	12,848,306	9,190,187	2
fSa1181	f	England, UK	SRR18750214	100	38	11,258,137	7,032,060	2
fSa1182	f	England, UK	SRX3888066	100	38	12,985,239	8,794,183	3
fSa526b	f	Austria	SRR18750223	50	39	16,379,376	9,891,666	2
fSa833d	f	Spain	SRX085166	50	33	61,309,304	43,659,901	4
fSa331a	f	Russia	SRR18750222	100	37	29,725,422	17,569,770	2
Sa283g	m	Belgium	SAMN08978280	50	37	47,027,899	32,592,752	5
Sa616	m	Germany	SRR18750221	100	38	29,391,799	19,664,547	2
Sa645	m	Germany	SRR18750220	100	38	26,958,655	18,848,991	2
Sa668a	m	Sweden	SRS3321307	50	39	42,129,557	28,584,622	5
Sa716	m	Greece	SRR18750219	100	38	27,814,452	18,245,053	2
Sa832	m	Spain	SRR18750218	100	38	24,783,472	16,373,356	2
Sa984	m	England, UK	SRX1100233	125	37	44,487,136	31,847,594	1
S. dioica								
fSd1175	f	England, UK	SRR18750217	100	38	16,190,466	11,861,668	2
fSd1176	f	England, UK	SRR18750216	100	38	15,870,430	11,131,695	2
fSd1177	f	England, UK	SRR18750213	100	38	9,238,845	5,602,414	2
fSd33b	f	England, UK	SRX3888067	100	38	11,298,605	7,734,327	3
fSd43a	f	Wales, UK	SRR18750212	50	39	22,738,642	13,995,221	2
fSd337a	f	England, UK	SRR18750229	50	39	18,541,178	11,147,501	2
fSd496a	f	Austria	SRR18750228	100	38	11,280,544	7,264,333	2
Sd554a	m	Austria	SRS3321308	50	39	11,572,078	8,742,918	5
Sd1168	m	England, UK	SRR18750227	100	38	31,407,131	21,104,671	2
Sd358a	m	Sweden	SRR18750226	50	39.5	40,147,695	24,692,255	2
Sd748a	m	Russia	SRS3321310	50	39	16,559,650	9,814,071	5
Sd868a	m	France	SRR18750225	50	39.5	14,898,008	9,241,493	2
Sd885	m	France	SRR18750224	50	39.5	19,122,033	11,593,651	2
Sd978	m	England, UK	SRS3321312	50	39.5	30,264,742	21,288,255	5
**Parents for *S. latifolia* cross: fSa985 x Sa984**								
**F1 progeny from fSa985 x mSa984 cross:**								
f101R	f	F1	SRX1100234	100	37	25,958,297	15,755,995	1
m101G	m	F1	SRX1100299	100	37	25,966,738	14,900,455	1
**F2 progeny from fSa985 x mSa984 cross** (F1: f101R x m101G):								

f108A1	f	F2	SRX1100300	100	37	26,697,947	15,571,249	[1]
f108B1	f	F2	SRX1100303	100	37	32,475,176	18,370,253	[1]
f108B	f	F2	SRX1100302	100	37	24,571,262	15,732,449	[1]
f108C	f	F2	SRX1100305	100	37	23,605,327	14,606,421	[1]
f108D	f	F2	SRX1100307	100	37	20,315,845	11,881,018	[1]
f108J1	f	F2	SRX1110504	100	37	28,869,725	16,594,465	[1]
f108J	f	F2	SRX1111588	100	37	25,281,056	15,250,824	[1]
f108K1	f	F2	SRX1107432	100	37	21,892,868	12,997,739	[1]
f108M1	f	F2	SRX1101617	100	37	22,336,786	13,521,921	[1]
f108N	f	F2	SRX1101237	100	37	26,168,869	16,648,249	[1]
f108o	f	F2	SRX1112915	100	37	30,397,583	18,106,782	[1]
f108R	f	F2	SRX1101234	100	37	23,309,105	13,912,773	[1]
f108S	f	F2	SRX1101233	100	37	31,024,291	19,221,533	[1]
f108U	f	F2	SRX1101231	100	37	24,676,474	14,512,495	[1]
f108Y	f	F2	SRX1101229	100	37	29,590,613	17,735,755	[1]
f108aa	f	F2	SRX1100301	100	37	10,177,030	6,275,173	[1]
f108bb	f	F2	SRX1100304	100	37	15,225,252	9,060,107	[1]
f108cc	f	F2	SRX1100306	100	37	14,008,379	7,938,544	[1]
f108dd	f	F2	SRX1100308	100	37	12,642,338	7,263,881	[1]
f108ee	f	F2	SRX1100576	100	37	13,231,930	7,831,724	[1]
f108ff	f	F2	SRX1100595	100	37	14,480,301	8,363,490	[1]
f108gg	f	F2	SRX1100610	100	37	15,624,099	8,858,318	[1]
f108LL	f	F2	SRX1109394	100	37	15,764,681	9,144,439	[1]
f108nn	f	F2	SRX1100617	100	37	11,692,591	6,948,081	[1]
f108pp	f	F2	SRX1100622	100	37	12,842,221	7,307,180	[1]
f108ss	f	F2	SRX1100628	100	37	12,685,738	7,735,355	[1]
f108uu	f	F2	SRX1100631	100	37	10,599,032	6,277,603	[1]
f108vv	f	F2	SRX1100632	100	37	13,979,886	8,163,205	[1]
f108ww	f	F2	SRX1100633	100	37	11,132,591	6,763,859	[1]
f108xx	f	F2	SRX1100634	100	37	12,588,592	7,307,577	[1]
f108zz	f	F2	SRX1100635	100	37	16,029,486	9,143,418	[1]
f108zzz	f	F2	SRX1100636	100	37	15,970,292	8,970,937	[1]
m108C1	m	F2	SRX1101071	100	37	26,062,118	14,734,464	[1]
m108E	m	F2	SRX1101064	100	37	31,341,740	18,375,009	[1]
m108F	m	F2	SRX1101058	100	37	29,393,157	16,708,677	[1]
m108G	m	F2	SRX1101031	100	37	33,794,797	19,141,171	[1]
m108H	m	F2	SRX1101026	100	37	29,409,381	16,838,995	[1]
m108I	m	F2	SRX1101023	100	37	42,104,306	25,541,222	[1]
m108K	m	F2	SRX1101021	100	37	23,122,563	13,370,262	[1]
m108P	m	F2	SRX1100944	100	37	24,538,451	15,443,548	[1]
m108T	m	F2	SRX1100941	100	37	26,173,820	16,288,050	[1]
m108W	m	F2	SRX1100940	100	37	27,878,524	15,729,868	[1]
m108X	m	F2	SRX1100917	100	37	34,727,348	20,289,710	[1]
m108Z	m	F2	SRX1100914	100	37	39,910,895	23,704,374	[1]
m108ff	m	F2	SRX1100678	100	37	13,104,259	7,832,814	[1]
m108hh	m	F2	SRX1100679	100	37	13,127,405	7,467,294	[1]

m108ii	m	F2	SRX1100680	100	37	9,722,070	5,933,636	1
m108kk	m	F2	SRX1100681	100	37	12,588,322	7,362,157	1
m108mm	m	F2	SRX1100682	100	37	12,735,206	7,607,082	1
m108oo	m	F2	SRX1100836	100	37	13,563,614	7,991,317	1
m108rr	m	F2	SRX1100845	100	37	14,628,163	8,872,861	1
m108tt	m	F2	SRX1100870	100	37	15,553,571	8,941,068	1
m108yy	m	F2	SRX1100873	100	37	13,441,320	8,138,061	1

References cited in Table S1

1 Papadopulos, A. S., Chester, M., Ridout, K. & Filatov, D. A. Rapid Y degeneration and dosage compensation in plant sex chromosomes. *Proc Natl Acad Sci U S A* **112**, 13021-13026, doi:10.1073/pnas.1508454112 (2015).

2 Filatov, D. A. Recent expansion of the non-recombining sex-linked region on *Silene latifolia* sex chromosomes. *J Evol Biol* **35**, 1696-1708, doi:10.1111/jeb.14063 (2022).

3 Krasovec, M., Nevado, B. & Filatov, D. A. A comparison of selective pressures in plant X-linked and autosomal genes. *Genes (Basel)* **9**, 234, doi:10.3390/genes9050234 (2018).

4 Chibalina, M. V. & Filatov, D. A. Plant Y chromosome degeneration is retarded by haploid purifying selection. *Curr Biol* **21**, 1475-1479, doi:10.1016/j.cub.2011.07.045 (2011).

5 Filatov, D. A. The two "rules of speciation" in species with young sex chromosomes. *Mol Ecol* **27**, 3799-3810, doi:10.1111/mec.14721 (2018).
